# Isolation of Genetically Tractable Most-Wanted Bacteria by Metaparental Mating

**DOI:** 10.1038/srep13282

**Published:** 2015-08-21

**Authors:** Páraic Ó Cuív, Wendy J. Smith, Sian Pottenger, Sriti Burman, Erin R. Shanahan, Mark Morrison

**Affiliations:** 1 CSIRO Preventative Health Flagship Research Program, Queensland, Australia; 2 The University of Queensland Diamantina Institute, The University of Queensland, Translational Research Institute, Queensland, Australia; 3 Department of Gastroenterology and Hepatology, Princess Alexandra Hospital, Queensland, Australia

## Abstract

Metagenomics has rapidly advanced our inventory and appreciation of the genetic potential inherent to the gut microbiome. However it is widely accepted that two key constraints to further genetic dissection of the gut microbiota and host-microbe interactions have been our inability to recover new isolates from the human gut, and the paucity of genetically tractable gut microbes. To address this challenge we developed a modular RP4 mobilisable recombinant vector system and an approach termed metaparental mating to support the rapid and directed isolation of genetically tractable fastidious gut bacteria. Using this approach we isolated transconjugants affiliated with *Clostridium* cluster IV (*Faecalibacterium* and *Oscillibacter* spp.), *Clostridium* cluster XI (*Anaerococcus*) and *Clostridium* XIVa (*Blautia* spp.) and group 2 ruminococci amongst others, and demonstrated that the recombinant vectors were stably maintained in their recipient hosts. By a similar approach we constructed fluorescently labelled bacterial transconjugants affiliated with *Clostridium* cluster IV (including *Flavonifractor* and *Pseudoflavonifractor* spp.), *Clostridium* XIVa (*Blautia* spp.) and *Clostridium* cluster XVIII (*Clostridium ramosum*) that expressed a flavin mononucleotide-based reporter gene (*evoglow-C-Bs2*). Our approach will advance the integration of bacterial genetics with metagenomics and realize new directions to support a more mechanistic dissection of host-microbe associations relevant to human health and disease.

Since their establishment, the International Human Microbiome Consortium, Human Microbiome Project (HMP)^1^ and MetaHIT^2^ have led efforts to provide (meta)genomic based insights into the representative microbial communities of the human body and that of representative microbial taxa with the expectations that this will provide new opportunities to better understand the role of the microbiota in health and disease. Despite the wealth of data and knowledge that has been generated by these initiatives much of the functionalities encoded in the microbiomes of the human body and other complex communities in nature remains cryptic. It is indisputable that techniques in bacterial genetics have been instrumental over many decades in deciphering the functional attributes and physiological characteristics of the microbial world but it is widely recognised that the development and application of these techniques with complex microbial communities has not kept pace with those of alternative metagenomic and deep DNA sequencing based technologies. Furthermore, while advances have been made in the isolation of “new” microbes via the use of genomic and/or metagenomic data^3^,^4^,^5^ bringing these microbiomes to life in the form of axenic or defined mixed cultures remains challenging.

With the notable exceptions of lactic acid bacteria and Gram negative bacteria assigned to the *Bacteroides* (e.g. *Bacteroides fragilis* and *Bacteroides vulgatus*^6^) and Proteobacteria (e.g. *Escherichia coli* and *Desulfovibrio piger*^7^), there is a paucity of cultured gut microbes known to be amenable to techniques in bacterial genetics which has placed a limit on our ability to interrogate and understand their functional potential, and their role in the gut ecosystem. Interestingly, (meta)genomic datasets have shown that the gut microbiota has been extensively shaped by lateral gene transfer^8^,^9^,^10^ and several studies have shown that gut microbes can exchange DNA by conjugative transfer *in situ*^11^,^12^,^13^. However to our knowledge no significant effort has been made to exploit these observations to recover genetically tractable fastidious gut microbes which if successful, would transform both the isolation and dissection of the microbiota that comprise these communities.

New approaches are needed that support the isolation and/or genetic dissection of key gut microbes as this will offer the best opportunities to conclusively link specific genetic factors with discrete biological activities. To address this challenge we developed an approach based on bacterial conjugation that supports the directed isolation of genetically tractable bacteria by a “metaparental” mating protocol using newly designed mobilisable vectors; and we used this approach to recover strains of key Firmicutes-affiliated bacteria from human stool. We chose to target bacteria from the Firmicutes as representatives from this phylum constitute a significant component of the human gut microbial core^14^,^15^,^16^, although they are poorly represented by cultured isolates and relatively few of the existing isolates have been assessed for their amenability to genetic characterization. We termed the conjugation of a vector from a donor strain to a mixed microbial community as a “metaparental mating” because it uses a single donor strain and numerous potential recipient strains in contrast to biparental mating (single donor and recipient strains) and triparental mating (single donor, helper, and recipient strains).

## Results

### The pEHR5 vectors are efficiently conjugated under aerobic and anaerobic conditions

**Figure 1:**
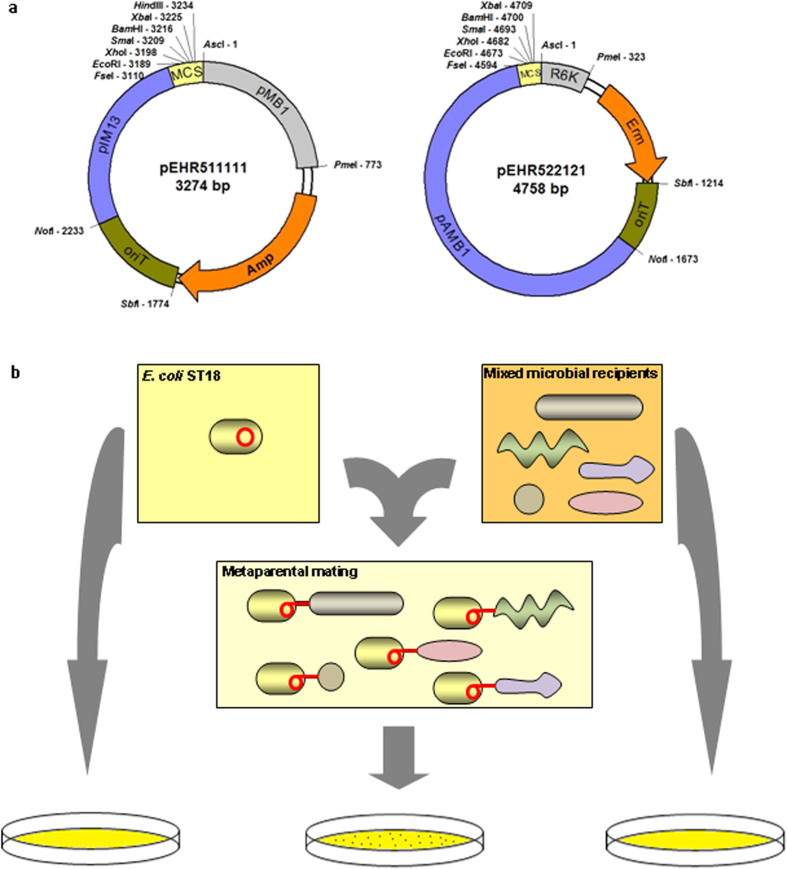
(**a**) The pEHR5 parent vector series with the five individual vector modules. The module associated 8-bp restriction enzyme sites are indicated in addition to unique sites from the MCS. The pEHR5 vector nomenclature follows the arrangement of the modules with the *E. coli* replicon module being the first module (1. ColE1 or 2. R6K modules) followed in order by the antibiotic resistance markers (1. Ampicillin (Amp) or 2. Erythromycin (Erm)), origin of transfer (1. *oriT*), non-*E. coli* origin of replication (1. pIM13 or 2. pAMβ1) and multiple cloning site (1. MCS). (**b**) Overview of the metaparental mating process. *E. coli* ST18 based vector donors are mixed with a culture containing numerous potential recipients derived from human stool. The metaparental mating is supported by spotting the cell mixture onto a nylon filter overlaid onto solid medium to facilitate the conjugative transfer of the vector. Transconjugants supporting stable vector replication are identified by plating on medium supplemented with antibiotics to which the vector confers resistance. The *E. coli* ST18 based vector donor and recipient cultures are processed identically in parallel.

We designed the small shuttle vector pEHR5 (***E***xtensible ***H***ost ***R***ange) so that it can be effectively conjugated from an *Escherichia coli* donor strain bearing the RP4 conjugative transfer system to naïve bacterial recipients (Fig. 1a). The pEHR5 based vectors were ultimately transferred to *E. coli* ST18^17^ which was used as the donor strain for all of the bacterial mating experiments. *E. coli* ST18 is a derivative of *E. coli* S17-1λ*pir*^18^ and carries a chromosomally encoded RP4 conjugative transfer system encoding all of the functions necessary for the efficient transfer of vectors bearing an *oriT* into amenable recipients. In traditional bi- and triparental matings an antibiotic to which the recipient is known to be resistant is typically used to counter select against the *E. coli* S17-1λ*pir* donor strain however, this strategy is not appropriate for metaparental matings as the antibiotic resistance susceptibilities of potential recipient strains may not be known. Instead, *E. coli* ST18 carries a deletion in *hemA* allowing efficient counter selection by the omission of δ-aminolevulinic acid from the growth medium and without the use of antibiotics.

First, the transfer efficiency of the vector pEHR511111, carrying an ampicillin resistance gene, to a newly isolated strain of *E. coli* (PC1101, recovered from a stool sample collected from a healthy human subject) was examined. Vector transfer efficiencies were high for both aerobic and anaerobic matings conducted on nylon filters (1.3 × 10^−2^ ± 8.51 × 10^−3^ and 1.0 × 10^−2^ ± 1.68 × 10^−3^ transconjugants/recipients, respectively) but were less so in microfuge tubes (1.8 × 10^−3^ ± 8.63 × 10^−4^ and 4.0 × 10^−4^ ± 1.73 × 10^−4^ transconjugants/recipients for aerobic and anaerobic conditions, respectively). As expected, pEHR511111 recovered from *E. coli* PC1101 (n = 12) showed similar mobility to vector prepared from *E. coli* DH5α as assessed by agarose gel electrophoresis indicating that that vector was stably maintained in the recipient following conjugation.

### Metaparental mating recovers genetically tractable bacteria from complex communities

We next applied the pEHR5 vectors to determine if genetically tractable Firmicutes-affiliated bacteria could be recovered by a metaparental mating approach from a microbial enrichment culture derived from human stool (Fig. 1b). We first assessed the diversity of the microbial enrichment culture produced using habitat simulating M10 medium and a stool sample collected from a healthy human adult. Serial dilutions of the enrichment culture were plated on M10 based medium and from the approximately 200 colonies recovered 12 were randomly selected and shown to be predominantly affiliated with the Bacteroidetes (75%), with lesser amounts of Proteobacteria (16.7%) and Firmicutes (8.3%) also present. The addition of erythromycin or chloramphenicol to M10 medium virtually eliminated outgrowth from the enrichment culture and based on these findings we chose to use a broad host range erythromycin resistance gene to construct the vectors pEHR512111 and pEHR512121 (bearing an erythromycin resistance marker^19^ and the Firmicute pIM13 and pAMβ1 origins of replication, respectively). We anticipated that the conjugative transfer of these vectors would result in the recovery of *E. coli* from the stool sample (via the *E. coli* pMB1 origin of replication) but also ensure the selective isolation of Firmicutes-affiliated transconjugants.

The metaparental matings were established anaerobically on nylon filters and the mixtures were subsequently spread on M10 based medium with and without added erythromycin. Individual donor and recipient cultures were similarly processed to confirm their inability to grow on medium lacking δ-aminolevulinic acid and in the presence of erythromycin, respectively. Following 7 days incubation, 2 × 10^−2^ pEHR512111 transconjugants/recipients and 3 × 10^−2^ pEHR512121 transconjugants/recipients were recovered. Colonies from both matings were randomly selected and their taxonomy was established by partial sequencing of the 16S rRNA (*rrs*) gene. The transconjugants characterized (n = 20) were pre-dominantly assigned to *Clostridium* cluster XIVa (*Blautia hathewayi*, *Blautia citroneae* and *Blautia bolteae*) and cluster XI (*Anaerococcus vaginalis*) with single transconjugants assigned to *Clostridium* cluster XVI (*Streptococcus pleomorphus*) and the Bacilli (*Paenibacillus macerans* and *Enterococcus* sp.) also recovered. As expected, some *E. coli* transconjugants were recovered in addition to the Firmicutes-affiliated transconjugants but notably, no Bacteroidetes affiliated isolates were recovered following the metaparental mating.

### Culture conditions affect the bacterial diversity recovered by metaparental mating

**Figure 2:**
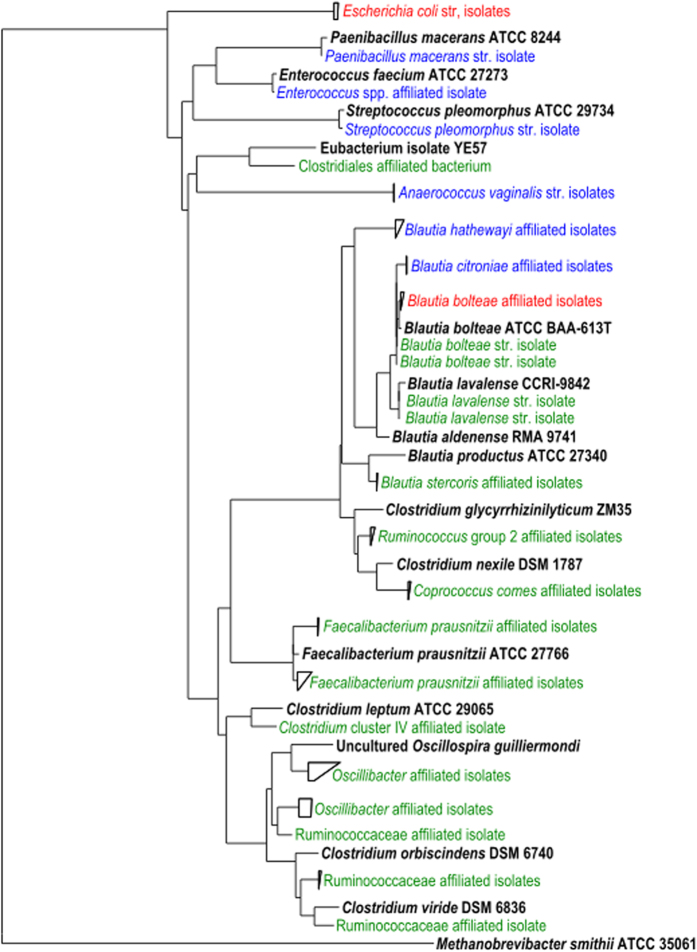
Phylogenetic analysis of the *rrs* gene of representative bacterial transconjugant isolates recovered on M10 and M2SC based media following metaparental mating. Isolates recovered on M10 and M2SC medium are indicated in blue and green typeface respectively while similar isolates recovered on both types of media are indicated in red typeface. The isolates were identified on the basis of partial sequencing of the *rrs* gene and the presence of the vector was confirmed by PCR and/or vector rescue. Representative microbial isolates and reference sequences are shown in bold black typeface.

As culture medium is often a key factor affecting the recovery of gut microbes we next repeated the metaparental mating using the same two vectors but instead used habitat simulating M2SC medium to produce the recipient population from the same parent stool sample. Although no discernible differences were observed in either the growth of the recipient population, or its conjugation efficiencies (7.8 × 10^−3^ pEHR512111 transconjugants/recipients and 1 × 10^−2^ pEHR512121 transconjugants/recipients), there was a substantial impact on the taxonomic profile of the transconjugants recovered (Fig. 2). From these matings, the partial *rrs* gene sequences showed the transconjugants characterized (n = 43) to be affiliated with *Clostridium* cluster XIVa (including, *B. bolteae*, *Blautia lavalense, Blautia stercoris*, and *Coprococcus comes*), *Clostridium* cluster IV (including *Faecalibacterium prausnitzii* and *Oscillibacter* spp) and the Proteobacteria (*E. coli*). Notably, we also recovered several “new” isolates affiliated with the Lachnospiraceae and Ruminococcaceae (Fig. 2). Interestingly, further analysis of the transconjugants by closed reference OTU picking revealed that isolates representing members of the HMP’s “most wanted” bacterial taxa^20^ and affiliated with the high priority Clostridiales taxon (OTU_998_V1V3), the medium priority *Oscillospira* taxon (OTU_640_V3V5, OTU_854_V3V5, OTU_957_V3V5 and OTU_1041_V1V3) and the low priority *Blautia producta* taxon (OTU_1270_V1V3) were also recovered. Based on these results, we conclude that the breadth of bacterial diversity recovered by metaparental mating can be significantly increased using recipient cultures that have been derived with different cultivation methods.

### Metaparental mating derived bacterial isolates carry pEHR5 vectors

**Figure 3:**
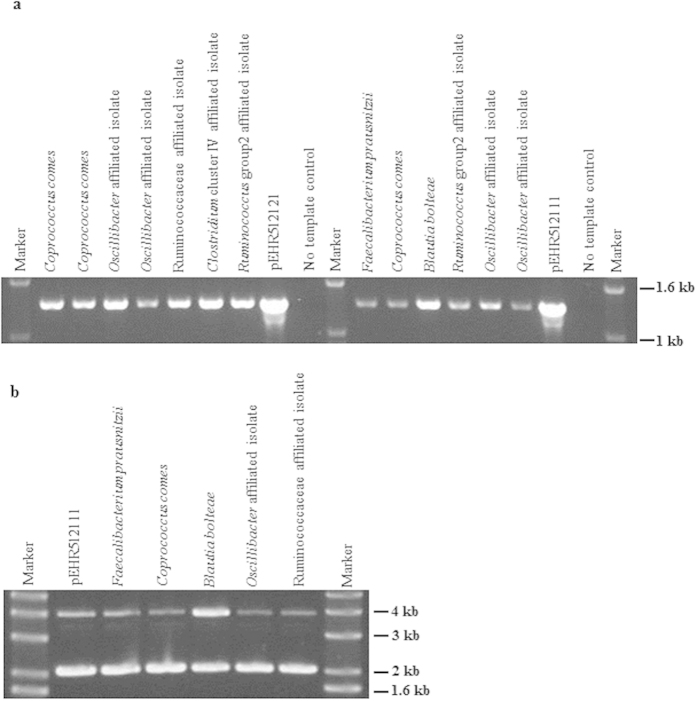
(**a**) Analysis of vector specific PCR products (*circa* 1.4 kb) amplified from putative transconjugants bearing recombinant vectors. The PCRs were performed on individual transconjugants isolated on M2SC medium with primers targeting the region carrying the erythromycin resistance gene and *oriT* modules. (**b**). Analysis of undigested pEHR512111 vectors (linear vector *circa* 3.2 kb) rescued from individual transconjugants strains isolated on M2SC medium and prepared from *E. coli* DH10B-T1^R^.

To confirm transfer of the pEHR5 based vectors Firmicutes-affiliated transconjugant strains were repeatedly sub-cultured from single colonies to ensure clonality and then the presence of the vectors was confirmed by PCR, using vector-specific primers targeting the erythromycin resistance gene and *oriT* modules of the vectors (Fig. 3a). Separately, we also recovered pEHR512111 vector DNA from some of the same transconjugant strains and used this DNA to transform *E. coli* DH10B-T1^R^, so as to confirm the structural integrity of the vectors (Fig. 3b). Interestingly, transformation of the recovered vector DNA into *E. coli* DH5α resulted in the identification of two different populations of transformants: one-bearing the intact vector and another shown by restriction digest analysis to contain vector with a deletion spanning the region encoding the pIM13 origin of replication and *oriT* module. We believe this deletion arises because the vector DNA is likely to have been methylated by its Firmicutes host, making it susceptible to the methylation-dependent restriction enzymes of *E. coli* DH5α, as has been previously observed^21^,^22^.

### Metaparental mating supports the construction of fluorescently labelled fastidious bacteria

**Figure 4:**
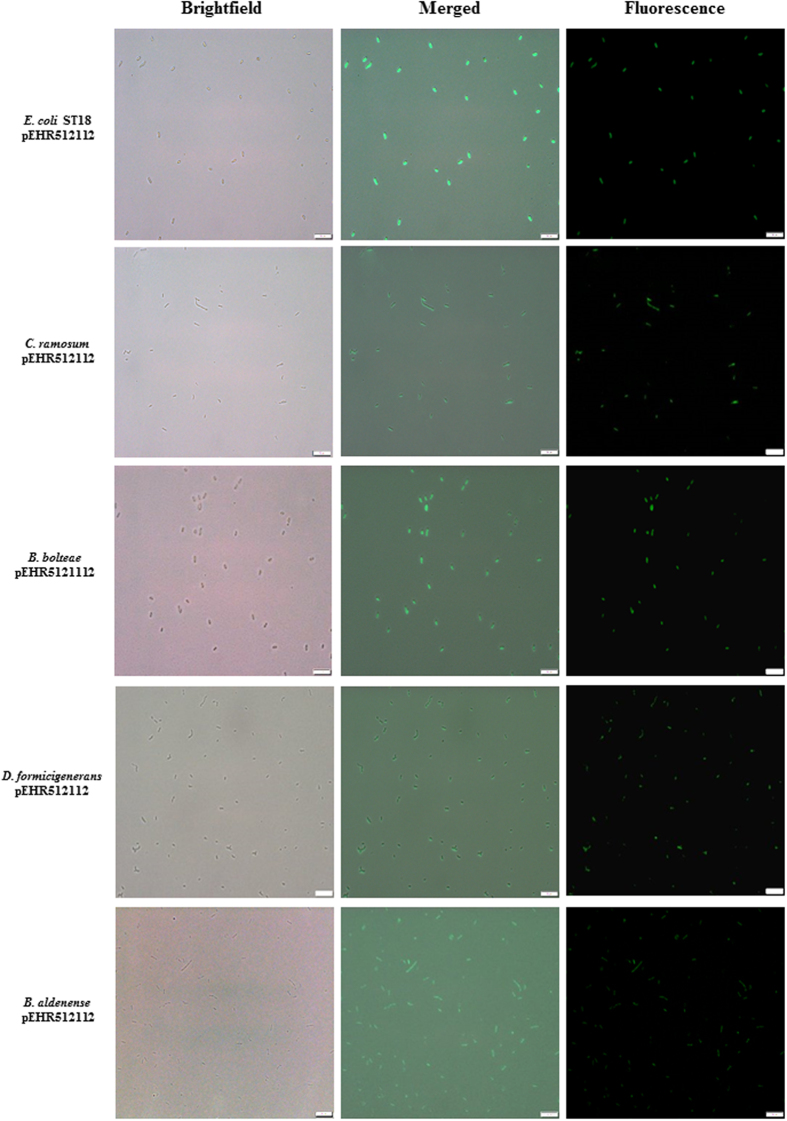
Analysis of transconjugants carrying pEHR512112 by fluorescence microscopy. The transconjugants were recovered and purified on M2SC based medium. Colonies were re-suspended in sterile anaerobic diluent and individual cells were visualised using an Olympus BX 63 microscope. Images were captured using the Olympus cellSens modular imaging software platform and processed using the ImageJ software package. A scale bar of 10 μm is included for reference.

To further validate our approach we constructed the vector pEHR512112 bearing the *evoglow-C-Bs2* gene under the control of the *Clostridium phytofermentans* cphy_3290 (*groES*) promoter and ribosome binding site. The *evoglow-C-Bs2* gene encodes a flavin mononucleotide-based fluorescent protein that functions under aerobic and anaerobic conditions and we anticipated that the *groES* promoter would result in constitutive expression of *evoglow-C-Bs2* in transconjugant hosts. The pEHR512112 vector was conjugated to a microbial enrichment produced using M2SC medium and a stool sample collected from a healthy human preadolescent child. By this approach we recovered fluorescent transconjugants (n = 27) that were affiliated with *Clostridium* cluster XIVa (including *B. bolteae*, *B. citroneae*, *Dorea formicigenerans*), *Clostridium* cluster IV (including *Flavonifractor plautii*, *Pseudoflavonifractor* sp.) and *Clostridium* cluster XVIII (*Clostridium ramosum*)(Fig. 4), and these included taxa similar to those recovered from the adult derived stool enrichment. We also recovered a non-Firmicutes affiliated transconjugant affiliated with the Actinobacterium *Eggerthella lenta*. Finally, from as few as 27 isolates we identified two isolates affiliated with the HMP’s most wanted low priority *Eubacterium* taxon (OTU_323_V3V5), and one isolate each affiliated with the low priority *Dorea* taxon (OTU_395_V3V5) and the low priority Lachnospiraceae taxon (OTU_472_V3V5).

## Discussion

The vast majority of gut microbes remain uncultured and recent efforts have used an increasingly broad range of culture conditions and high-throughput approaches to increase the number of axenic isolates from the gut microbiota^23^,^24^. However, the vast majority of cultured gut bacteria are not known to be manipulable by techniques in bacterial genetics and the extent of genetically tractable bacteria in the human gut remains unknown. This currently represents a major impediment to our ability to genetically dissect the functional capabilities of the microbiota and in this study we examined whether bacterial conjugation could be used to recover from complex gut microbial communities genetically tractable gut bacteria affiliated with known and novel microbial lineages within the Firmicutes. To address this issue we used the promiscuous RP4 conjugative transfer system to recover genetically tractable gut bacteria as we believed that it possessed three key advantages over alternative approaches (e.g. electroporation, sonoporation). First, the recipient cultures do not typically need to be pre-treated prior to conjugation and the metaparental mating can be easily performed under physiologically relevant (e.g. anaerobic) conditions. Second, the single-stranded DNA transferred during the conjugation process may be less susceptible to digestion by the recipient host restriction modification systems^25^. Third, our approach combines bacterial isolation with what is effectively a high-throughput screen for genetically tractable strains; in contrast to existing methods where the genetically tractability of isolates is assessed on an individual basis. Our results demonstrate that bacterial conjugation can be used to recover a broad range of genetically tractable gut bacteria. To our knowledge, this is the first report of an efficient method for the recovery of genetically tractable isolates, including members of the core microbiota, from the human gut.

In this study we specifically targeted Firmicutes-affiliated bacteria via the vector origin of replication although we used relatively growth permissive culture conditions as the nutritional requirements of many of these bacteria are unknown. By this approach we recovered similar taxa from our adult and pre-adolescent derived microbial enrichments in addition to a broad range of sample specific taxa, which is consistent with the presence of an abundant healthy core microbiota that is shared between individuals and a subject specific accessory microbiota^16^,^17^. Interestingly, metaparental mating and vectors carrying Firmicutes derived origins of replication were capable of selectively recovering Firmicutes-affiliated bacteria, including from a Bacteroidetes dominated M10 medium derived enrichment culture. We also observed differences in the microbes recovered using M10 and M2SC although both types of medium are broadly similar in composition suggesting that the use of a broader selection of microbiological media for the generation of the enrichment cultures and/or for the recovery of the transconjugants on agar plates would have further increased the diversity of gut microbes isolated^23^,^24^. The recovery of sub-dominant transconjugants from a mating mix could additionally be enhanced by counter selecting specific bacterial groups by nutrient limitation (e.g. hemin and/or vitamin K limitation to inhibit the growth of *Bacteroides* spp. and related taxa), the addition of growth inhibiting chemicals (e.g. sodium azide or bile salts to inhibit the growth of Gram negative and positive bacteria respectively) or antibiotic selection (e.g. vancoymycin and/or teicoplanin to inhibit the growth of Gram positive bacteria and polymyxin and/or nalidixic acid to inhibit the growth of Gram negative bacteria). The direct recovery of microbes from stool for use as recipients in a metaparental mating could also further maximize the recovery of genetically diverse isolates particularly as the production of enrichment cultures can lead to medium specific enrichment biases^26^,^27^.

The metaparental mating approach also recovered several isolates representing some of the Human Microbiome Project’s “most-wanted” bacterial taxa^20^ in addition to other taxa with relevance to human health. For instance, the metaparental mating methods have produced some of the first human-derived isolates of *Oscillibacter* spp., which are widely believed to be a member of the healthy core microbiota resident in the human gut^15^, but that showed significant variations in abundance in a cross-sectional study of the stool microbiota from healthy and Crohn’s disease subjects^28^. Additionally, some of the isolates recovered are phylogentically affiliated with taxa considered to be immunomodulatory^29^,^30^ or to be comprised of assacharolytic bacteria^31^,^32^, further broadening the functional diversity of the isolates available for gut microbiota studies. We cannot exclude the possibility that some of these (most-wanted) taxa were abundant in our microbial enrichments and qPCR/*rrs* profiling based pre-screening could help identify recipient cultures enriched with target taxa and expedite their recovery as axenic cultures.

The construction of the pEHR5 vector series and the development of the metaparental mating approach are critical steps in the development of new methodologies that will support the functional characterisation of fastidious gut bacteria. The human gut is characterized by a distinct biogeography^33^,^34^,^35^ and our construction of fluorescently labelled gut bacteria may provide new insights into their spatial distribution and functional role. For instance, we isolated fluorescently labelled bacteria affiliated with taxa known to induce regulatory T cells^30^,^36^ and to modulate expression of glucose transporter 2 in the jejunum and fatty acid translocase in the ileum^37^ of mice. Critical aspects of the mechanism(s) by which these bacteria exert their effects remain to be determined and identifying the specific gut niches colonised may provide new insights into their interactions with the host. We anticipate that the pEHR5 vectors will facilitate the characterization of bacterial isolates by forward and reverse genetic approaches and in support of these objectives the pEHR5 vectors are currently being adapted to carry a retrohoming element (the Ll.ltrB intron^38^) and to function as suicide vectors for targeted mutagenesis; and we anticipate that they can also be adapted to carry transposable elements (e.g. Himar) for random mutagenesis.

In conclusion, the metaparental mating approach and pEHR5 vector series described here offers a rapid and powerful way to not only recover potentially genetically tractable isolates of gut bacteria but to also produce axenic cultures of those microbes affiliated with some of the “most-wanted” taxa that have remained elusive to cultivation. The pEHR5 vectors are also effectively conjugated in a microfuge tube based mating suggesting that this process could be automated and scaled via multi-well plates The method is an excellent complement of recent efforts to improve functional (meta)genomic approaches that include broadening the host range of metagenomic cloning vectors^39^,^40^ and increasing the through-put of bacterial protein-protein interaction mapping for protein function discovery^41^; and offers new opportunities to dissect host-microbe interactions via genetic manipulation of the host and/or the microbiota. The host range of the pEHR5 vectors can be readily extended by using other origins of replication and resistance markers and we anticipate that metaparental mating can be adapted to recover a broader cross-section of genetically tractable bacteria representing known and “new” lineages resident with the human body as well as other complex environments. For these reasons we believe that it is a valuable addition to the suite of methods that are currently being used to better understand the gut ecosystem and that it will help bring the microbiome to life.

## Materials and Methods

### Bacterial strains and culture conditions

The *E. coli* ST18 strain was used for the bi- and metaparental matings while the *E. coli* DH5α and *E. coli* DH10B-T1^R^ (Invitrogen) strains were used for vector transformation. Competent *E. coli* ST18 cells were prepared by the rubidium chloride method^42^ while competent *E. coli* DH5α and *E. coli* DH10B-T1^R^ cells were purchased from Invitrogen. *E. coli* was routinely cultured using LB medium. Ampicillin, erythromycin and δ-aminolevulinic acid were used at a final concentration of 100 μg/ml as appropriate. Recipient cultures were derived from stool samples collected from a healthy human adult and a healthy human preadolescent child with no history of gastrointestinal disorders following informed consent and ethical guidelines approved by the Commonwealth Scientific and Industrial Research Organisation and The University of Queensland. None of the volunteers were consuming probiotics and had not consumed antibiotics in the three months prior to the sample collection. To ensure experimental reproducibility anaerobic glycerol stocks of the recipient culture was prepared as follows: Approximately 100 μl of the anaerobic stool glycerol stock was used to inoculate anaerobic starch free M10 medium^43^ supplemented with 30% (v/v) clarified bovine rumen fluid or glucose free M2GSC medium^44^ (M2SC). We chose to use M10 and M2SC as they are considered to be habitat stimulating media and they have been shown to support the growth of a diverse range of gut microbes^44^,^45^. Each culture was incubated horizontally at 37 °C for 24 hours with agitation at 60 rpm. Then three ml of the culture was mixed with three ml of sterile anaerobic glycerol and stored at −80 °C. Recipient cultures for metaparental mating were subsequently prepared by transferring 50 μl of the glycerol stock to M10 or M2SC based medium and incubating as described above. A Coy vinyl anaerobic chamber with an oxygen free carbon dioxide/hydrogen (95% CO_2_:5% H_2_) atmosphere was used to process the recipient cultures and to perform the metaparental mating experiments. Laboratory chemicals were purchased from Sigma Aldrich.

### DNA manipulation and vector construction

Each pEHR5 vector contains five ordered modules: (i) an *E. coli* origin of replication to support vector replication in the donor strain, (ii) an antibiotic resistance marker gene to support vector selection in the donor and recipient strains, (iii) an *oriT* site to support RP4 based conjugative transfer, (iv) a non *E. coli* origin of replication, and; (v) a multiple cloning site (MCS) (Fig. 1a). Each individual vector module is flanked by unique sites for 8-bp cutting restriction enzymes thus supporting the easy exchange of individual modules with replacement modules bearing compatible restriction sites and we have used a standard nomenclature to define the vector constructs (see Fig. 1a). The ampicillin resistance gene from pUC19 was PCR amplified with primers (P_f_: 5′-GTT TAA ACA GGT GGC ACT TTT CGG GGA AAT GTG C; P_r_: 5′-CCT GCA GGT TAC CAA TGC TTA ATC AG) that introduced PmeI and SbfI restriction sites into the product. Separately, the *oriT* determinant from pJQ200sk + was PCR amplified with primers (P_f_: 5′-CCT GCA GGC GCA AAA AAC CCC GCT TCG GCG GGG TTT TTT CGC TTC CTG CAT TTG CCT GTT TCC AGT C; P_r_: 5′-GCG GCC GCC CTC AAT CGC TCT TCG TTC G) that introduced a unique SbfI restriction site and transcriptional terminator; and NotI restriction site respectively. The ampicillin resistance gene and *oriT* determinant were then SbfI digested, subject to intermolecular ligation and then PCR amplified to yield a single Amp-*oriT* product. The pIM13 origin of replication from pGusA^46^ was PCR amplified with primers (P_f_: 5′-GGC CGG CCC TTA ATC GCA TTT CAT AGA T; P_r_: 5′-GCG GCC GCG CAT TCA CTT CTT TTC TAT A) that introduced unique NotI and FseI restriction sites. Then the Amp-*oriT* and pIM13 products were digested with NotI, subject to intermolecular ligation and then PCR amplified to yield an Amp-*oriT*-pIM13product. Separately, the pMB1 origin of replication from pUC19 was PCR amplified with primers (P_f_: 5′-GGC GCG CCT TCG TTC CAC TGA GCG TC; P_r_: 5′-GGT ACC TTA AAT AAT TAA CTT AAA) that introduced unique AscI and PmeI restriction sites. The MCS fragment was synthesised by IDT Technologies Inc. The pEHR511111 vector was ultimately constructed following a three way ligation between the Amp-*oriT*-pIM13, pMB1 and MCS fragments. The pEHR522121 vector was constructed by PCR amplifying the erythromycin resistance gene (P_f_: 5′-GTT TAA ACG ATC TAC GCA GAT AAA TAA ATA CG; P_r_: 5′-CCT GCA GGT TAT TTC CTC CCG TTA AAT A) and the pAMβ1 origin of replication (P_f_: 5′-GCG GCC GCT ATT TAA TCA CTT TGA CTA; P_r_: 5′-GGC CGG CCC CAA GAA TTA GAA ATG AGT A) from pQexp^38^; and the R6K origin of replication from pUTmini-Tn5km^47^ (P_f_: 5′-GGC GCG CCA TCC CTG GCT TGT TGT CCA C; P_r_: 5′-GTT TAA ACG AAG ATC AGC AGT TCA ACC TG) and replacing the ampicillin resistance gene, pIM13 origin of replication and pMB1 origin of replication respectively of pEHR511111. To construct pEHR512112 the *C. phytofermentans* ISDg Cphy_3290 (*groES*) promoter region (chromosomal co-ordinates 4007675–4007501) was synthesised by Life Technologies Inc. The promoter region was subsequently amplified with primers (P_f_: 5′-GAT CGA ATT CAA GTG AGA ATA CAA GAG TAT AC; P_r_: 5′-GAT CTC TAG ATT CCT CCT TTA ATA CTG TAT C) that introduced unique EcoRI and XbaI restriction sites. Separately, the *evoglow-C-Bs2* gene from the recombinant plasmid pGlow-C-Bs-2 (Jena Bioscience GmbH) was amplified with a primer (P_f_: 5′-CAA ACC TAC CAT AAG GAG AAT CTA GAA TGG CAA GTT TTC AAA GTT T) that introduced a unique XbaI site and a primer (P_r_: 5′-GAT CAA GCT TTT ATT CAA GGA GCT TTT CAT ATT C) that moved an internal HindIII site to the 3′ end of the gene. XbaI digested Cphy_3290 promoter and *evoglow-C-Bs2* products were ligated and amplified using the Cphy_3290 forward primer and the *evoglow-C-Bs2* reverse primer. The fusion product was digested with EcoRI and HindIII and ultimately cloned into the pEHR512111 vector. Molecular enzymes were purchased from New England Biolabs and all of the vectors were confirmed by restriction digest analysis.

### Bi- and metaparental matings

*E. coli* ST18 donors bearing pEHR5 series vectors were grown in LB broth supplemented with δ-aminolevulinic acid and erythromycin or ampicillin; while the recipient culture was grown in the appropriate broth; at 37 °C. Cultures grown in the presence of antibiotics were washed thrice with sterile diluent and the donor and recipient cultures were then mixed in a ratio of 4:1 respectively on the basis of the OD_600_ of the respective cultures and the cell mixture was centrifuged at 13,000 g for 2 minutes. Individual donor and recipient cultures were similarly processed and the cell pellets were then spotted onto a nylon filter overlaid on an agar plate and incubated at 37 °C. Microfuge tube based matings were performed in a similar manner except that the cell pellets were incubated in microfuge tubes at 37 °C. The cell pellets were suspended in sterile anaerobic diluent and dilutions were spread on medium with and without erythromycin, and incubated at 37 °C until colonies were clearly visible.

### Culture dependent analysis and transconjugant characterization

For the culture dependent analysis the diversity of microbes recovered on M10 or M2SC medium was initially assessed on the basis of colony and/or cell morphology. For naïve microbes individual colonies were randomly picked and subject to PCR analysis by colony based PCR and/or by preparing DNA by the RBB + C method^48^ for hard to lyse cells, as determined by the ability to successfully PCR amplify the *rrs* gene. The identity of the individual isolates was determined by PCR amplification of the *rrs* gene using the primers 27F and 1492R^49^ as previously described by Ó Cuív, *et al.*^50^. Putative transconjugants were similarly assessed except that they were purified by streaking individual colonies up to three times on medium containing erythromycin to ensure clonality. Amplified *rrs* genes were partially sequenced using the primer 907R^49^ and the sequences were aligned against the core set of aligned *rrs* sequences at the RDP 10 website (rdp.cme.msu.edu) and the isolates were tentatively identified using the SeqMatch and/or Classifier functions^51^. Briefly, the sequences were initially examined using the SeqMatch function and taxonomy was assigned to the species level if the isolates displayed ≥97% sequence identify to type strains. In instances where a sequence exhibited ≥97% sequence identity to several type strains then the taxonomy was assigned to the type strain with the greatest sequence identity. Isolates exhibiting <97% similarity to type strains were further examined using the Classifier function and taxonomy was assigned to the lowest taxonomic level that achieved an ≥80% bootstrap value. Transconjugants displaying similarity to the HMP’s most wanted taxa were identified by clustering the transconjugant and HMP associated sequences using the closed reference OTU picking protocol implemented through QIIME v1.8.0^52^ and using a 97% similarity threshold. This approach allows non- and partially-overlapping amplicons to be clustered against a reference sequence set that spans the amplicons of interest. The taxonomic clusters were then examined to identify transconjugants affiliated with the HMP’s most wanted high priority, medium priority and low priority taxa.

The presence of the pEHR5 vectors in transconjugants was confirmed by PCR with primers targeting the vector region spanning the erythromycin resistance gene and the *oriT* modules. Vector rescue experiments were performed by recovering vector DNA essentially as described by Birnboim *et al.*^53^, and transforming it into *E. coli*. The structural integrity of the recovered vectors was determined by assessing the mobility of the undigested vector by agarose gel electrophoresis. Vectors with putative deletions were further characterized by restriction digest analysis using the 8 bp cutting restriction enzymes flanking the individual vector modules. Transconjugants carrying pEHR512112 were examined using an Olympus BX 63 microscope fitted with a DP80 camera, Xcite LED light source and fluorescence filter cube U-FBN (excitation 470–495 nm, emission 510 nm). Images were captured using the Olympus cellSens modular imaging software platform and processed using the ImageJ software package (http://imagej.nih.gov/ij/). To construct phylogenetic trees the *rrs* gene sequences were aligned using the QIIME v1.8.0 implementation of PyNAST and the alignment was then imported into ARB v5.5^54^. Next, the hypervariable regions of the aligned sequences were masked with the lanemaskPH filter and the sequences were added to the database tree using the ARB-parsimony tool. The alignment of the transconjugant sequences was refined as necessary and the database tree was then updated and a phylogenetic tree displaying the transconjugant derived sequences and select reference sequences was produced.

## Additional Information

**How to cite this article**: Ó Cuív, P. *et al.* Isolation of Genetically Tractable Most-Wanted Bacteria by Metaparental Mating. *Sci. Rep.* **5**, 13282; doi: 10.1038/srep13282 (2015).
